# Cytotoxic Potential of Denture Adhesives on Human Fibroblasts—In Vitro Study

**DOI:** 10.3390/ma15041583

**Published:** 2022-02-20

**Authors:** Ewa Sobolewska, Piotr Makowiecki, Justyna Drozdowska, Ireneusz Dziuba, Alicja Nowicka, Marzena Wyganowska-Świątkowska, Joanna Janiszewska-Olszowska, Katarzyna Grocholewicz

**Affiliations:** 1 Department of Dental Prosthetics, Pomeranian Medical University, 70-111 Szczecin, Poland; ewa.sobolewska@pum.edu.pl; 2 Department of Radiology, Pomeranian Medical University, 70-111 Szczecin, Poland; piotr.makowiecki@pum.edu.pl; 3 Department of Interdisciplinary Dentistry, Pomeranian Medical University, 70-111 Szczecin, Poland; justyna.drozdowska@pum.edu.pl (J.D.); jjo@pum.edu.pl (J.J.-O.); 4 Faculty of Medicine, University of Technology, 40-555 Katowice, Poland; mmid@wp.pl; 5 Faculty of Medicine, Collegium Medicum, Cardinal Stefan Wyszyński University in Warsaw, 01-815 Warsaw, Poland; 6 Department of Conservative Dentistry and Endodontics, Pomeranian Medical University, 70-111 Szczecin, Poland; alicja.nowicka@pum.edu.pl; 7 Department of Oral Surgery and Periodontology, Poznan University of Medical Sciences, 60-812 Poznan, Poland; wyganowska@ump.edu.pl

**Keywords:** dentures, denture adhesives, human fibroblasts, cytotoxicity

## Abstract

(1) In recent years, there has been a significant increase in the availability of denture adhesives for stabilizing removable dentures. The aim of the present study was to assess the cytotoxicity of three denture adhesives on human fibroblasts. (2) Methods: Three denture adhesives were analyzed. Fibroblast cultures were established for the study and control groups in order to assess the incidence of necrosis and to evaluate the microscopic intracellular alterations induced. Following incubation with (study groups) or without adhesives (control group), trypan blue dye exclusion assay was used to determine the number of viable and/or dead cells. Microscopic specimens were stained with haematoxylin and eosin, scanned, digitally processed and then analyzed by a histopathologist. (3) Results: All three denture adhesives analyzed demonstrated various toxic effects in vitro on human fibroblast: quantitative evaluation—45.87–61.13% reduction of cell viability (*p* = 0.0001) and slight to moderate cytotoxicity in qualitative evaluation. (4) Conclusions: Denture adhesive creams demonstrated a toxic effect on human fibroblasts in vitro in quantitative and qualitative evaluation. In vivo observations are needed to find out if denture adhesives present a cytotoxic effect in patients.

## 1. Introduction

Epidemiological data indicate a continuous increase in the number of edentulous patients. It has been attributed to elongation of global average life expectancy [1,2,3]. Prosthetic rehabilitation of edentulous patients is difficult and requires knowledge and experience, both from dentists and dental technicians. Despite considerable advances in the field of prosthodontics, conventional complete dentures are still the most popular prosthetic restorations in edentulous patients [4]. Significant bone resorption following teeth extractions deteriorates the clinical conditions for satisfactory denture retention and stability; retention and stability clearly decrease after several years [5,6]. Efforts have been made to develop a material for dental prostheses with the best functional properties [7]. Retention of dentures can be improved by using denture adhesives or relining dentures. Properly used denture adhesives can improve the retention and stability of prosthetic restorations and prevent food residue accumulation under the denture [6,8,9,10,11,12]. 

In recent years, there has been a significant increase in the availability of adhesives for stabilizing removable dentures. The study of Okazaki et al. showed that 19% of denture wearers use denture adhesives [13]. Most denture adhesives contain non-toxic polymers of carboxymethyl cellulose [14]. All creams that improve the stability of dentures also contain swelling agents, such as karaya gum, Arabic gum, tragacanth gum, gelatin, pectin, methylcellulose, hydroxyethylcellulose, synthetic polyethylene polymers and others. Another group of ingredients are antibacterial and antifungal agents: sodium borate, hexachlorophene and polyhydroxybenzoate [15,16]. Adhesives are thus compound products; their use exerts not only a local effect on the oral mucosa, but also may influence the general health [17,18,19]. Ingredients of adhesives (e.g., formaldehyde) may produce allergenic and cytotoxic effects [20,21,22]. Another negative feature of denture adhesives is their low pH (5.5 on average), which is capable of dissolving enamel hydroxyapatites in the remaining dentition [23]. Denture adhesives are often used for an extended time period, which causes excessive pressure on the denture base and consequently its progressive wear. This may be a potential factor causing pathologies of the soft tissues [24]. In the leaflets for adhesive creams, manufacturers recommend that they be applied pointwise by squeezing out strips a few millimeters long from the tube. However, patients usually do not follow these recommendations and use too much of these materials. Considering all these problems associated with the use of denture adhesives, especially of formaldehyde content, there is a justified need for testing their cytotoxicity, irrespective of the data provided by their manufacturers. 

Fibroblasts, the main group of connective tissue cells, are a heterogeneous group of cells, which, despite numerous similarities in structure and function, are characterized by significant differentiation depending on the anatomical location of the connective tissue, but those in the face and oral cavity are derived from the neural crest. There are also differences in fibroblasts isolated from healthy tissue and granulation tissue [25,26,27,28,29,30,31]. An important feature depending on the source of fibroblasts used in experimental studies is the rate of proliferation. Tooth pulp as an immature gelatinous tissue is rich in fibroblasts capable of rapid multiplication. 

The aim of the present study was to evaluate the cytotoxicity of three denture adhesives on human fibroblasts and to compare the effect of the analyzed products.

## 2. Materials and Methods

### 2.1. Harvesting Fibroblasts 

Fibroblasts were harvested from the pulp of 15 healthy (non-pathologically damaged) teeth extracted for orthodontic indications. All the patients involved were informed about the research project and signed an informed consent form according to guidelines from the Declaration of Helsinki. The study was approved by the Bioethics Committee of Pomeranian Medical University in Szczecin (Decision Reference No. KB-0012/05/13). Immediately after tooth extraction (up to 10 min) the pulp chamber was opened using a ball-shaped diamond drill in an air turbine head with water cooling. The pulp was removed using sterile root canal broaches and immediately suspended in Roswell Park Memorial Institute (RPMI) 1640 Medium (Sigma-Aldrich, St. Louis, MO, USA) supplemented with 20% fetal bovine serum (FBS; Sigma-Aldrich). 

### 2.2. Fibroblast Cultures

The extracted dental pulp was homogenized and the fibroblast cultures were established in tissue culture flasks (Sarstedt Inc., Newton, MA, USA). Cells were cultured in RPMI 1640 Medium supplemented with 20% FBS (Biological Industries, Beit-Haemek, Izrael) in an incubator under standard conditions (48 h, 37 °C, CO_2_ 5%, relative humidity 99.6%). Fibroblast cultures for the study and control groups were prepared in the Laboratory of Cell and Tissue Culture, Department of Genetics and Pathomorphology, Pomeranian Medical University in Szczecin. The culture of fibroblasts from tooth no. 1 presented abnormal growth of cells, probably caused by incorrect handling of biological material (pulp) before placing it in the transport medium. Thus, the number of cultures was 14, and each culture was supplemented with tested denture adhesives. 

### 2.3. Quantitative Evaluation

Three denture adhesives, commercially available in Poland, were tested. Their manufactures and compositions are presented in Table 1. It is visible that two of the adhesives tested (COREGA Extra Strong and PROTEFIX) do not contain zinc salts opposite to the other one (BLEND-A-DENT Plus). The composition of COREGA Extra Strong and PROTEFIX is very similar but not identical.

The assay was conducted according to the following procedure: 0.5 mL of each tested adhesive was placed in a Petri dish with 3 mL RPMI 1640 Medium supplemented with 20% FBS to obtain a solution. The Petri dishes were then placed in an incubator and kept for 5 days under standard culture conditions. After 5 days the solution was transferred to 96-well tissue culture plates (Sarstedt Inc., Newton, MA, USA). Each denture adhesive was placed into 3 wells (study groups), and one well was filled with a pure medium (as a negative control) to be used as the control group (K). Cultures of fibroblasts were established in media prepared this way by placing about 100,000 cells from the first passage. Culture plates were moved to the incubator set at standard parameters and incubated for 72 h. After this time, trypan blue dye exclusion assay was used to determine the number of viable and/or dead cells. Trypan blue is a ~960 Daltons molecule, which is cell membrane impermeable and therefore only enters cells with compromised membranes. Upon entry into the cell, trypan blue binds to intracellular proteins thereby rendering the cells a bluish color. The trypan blue exclusion assay allows for a direct identification and enumeration of live (unstained) and dead (blue) cells in a given population. For that the cell culture was stained with 0.4% Trypan Blue solution (Sigma-Aldrich, St. Louis, MI, USA). Then, viable and necrotic fibroblasts were counted using an Axiovert 25 inverted transmitted light microscope (Carl-Zeiss, Jena, Germany) and a glass hemocytometer. Trypan blue was added to an Eppendorf tube with 100 µL of cells 400 µL 0.4% (final concentration 0.32%). Using a pipette, 100 µL of trypan blue-treated cell suspension was applied to the hemocytometer. Viable (unstained) and necrotic (blue stained) cells were counted in all 16 squares under the microscope with a 100× magnification. Cell counting was performed 3 times for each well. Counting was carried out by the same person, unfamiliar with the tested materials. The results from all wells for a given adhesive were summed up and averaged. For the control culture, counting of viable and necrotic cells was carried out in the same way, using a glass hemocytometer, but the cells were taken from three different places of the well. The results were also summed up and averaged. 

In order to assess the incidence of necrosis after in vitro cell culture, an AI (apoptotic index) according to Prieto was used [32]. It is calculated by dividing the percentage of apoptotic cells by the total percentage of cells in the sample. In the present study the index was modified by using it to calculate the percentage of necrotic cells.

The results were subjected to statistical analysis. Statistical analysis was performed using STATA 11 software. All continuous variables were verified for distribution normality using a Shapiro-Wilk test. Statistical significance of differences between two groups was analyzed using a Mann-Whitney test. To investigate the relationship between two variables a chi^2^ Pearson test and Spearman’s rank correlation test were used. The level of statistical significance was set at α = 0.05. The risk of cell necrosis was expressed as an odds ratio (OR) at 95% confidence interval (CI). Differences were considered significant if the level of significance was *α* = 0.05. 

### 2.4. Qualitative Evaluation

In parallel, fibroblasts from dental pulp were cultured in order to assess the microscopic changes induced in the cells and to prepare microscope slides of cells damaged by the tested adhesives. Microscope slides were placed on Petri dishes with fibroblasts from the first passage cultured in a mixture of RPMI 1640 Medium, 20% FBS and different denture adhesives. These were the study groups. The same procedure was followed to establish the control group (K), which was a fibroblast culture in pure RPMI 1640 Medium. The cultures were placed in an incubator and kept for 72 h under standard conditions. After incubation the fibroblasts attached to the slides were stained with haematoxylin (Haematoxylin, Fluka, Switzerland) and eosin (Eosin Yellowish, Loba Chemie, Mumbai, India) in a standard procedure (HE). Prepared microscopic slides were assessed using a light standard laboratory microscope (Olympus BX 43, Olympus Corporation, Tokyo, Japan) with magnification of 100× and 200×. Then, the slides were scanned using an Aperio CS2 pathology scanner (Leica Microsystems, Wetzlar, Germany) to take a photograph at a magnification of 100× and 200×. The histopathologist did now know the materials were being assessed.

### 2.5. Determination of Cytotoxicity

The cytotoxic effect was evaluated quantitatively and qualitatively according to INTERNATIONAL STANDARD ISO 10993-5:2009(E) [33]. According to this standard, reduction of cell viability by more than 30% is considered a cytotoxic effect. Qualitative morphological grading of cytotoxicity is based on assessing of general morphology, vacuolization, detachment, cell lysis and membrane integrity and expressed on a five-point scale.

Comparison of the necrotic effect of the adhesives on fibroblasts made it possible to divide the creams into three classes and identify products which induced the lowest (CLASS 1), moderate (CLASS 2) or the highest (CLASS 3) number of necrotic cells. CLASS 1 included all cases of the tested cream in which the number of necrotic cells was lower than that of both samples of the other two materials. CLASS 3 included all the cases of the tested cream in which the number of necrotic cells was higher than that of the samples of other materials. If the number of necrotic cells in the sample with the tested material was smaller than in the sample with the second material and at the same time higher than in the sample with the third material, it was classified as CLASS 2.

## 3. Results

### 3.1. Qantitative Evaluation of Cytotoxic Effect

Table 2 presents descriptive statistics for the value of necrotic fibroblasts in the study and control groups expressed in %. For all tested materials, a significantly higher percentage of necrotic cells was found compared to the control cultures (*p* < 0.0001). The highest percentage of necrotic cells was observed in culture supplemented with COREGA Extra Strong. Although COREGA Extra Strong and PROTEFIX have a similar composition, their necrotic effect on pulp fibroblast is different. Quantitative evaluation showed a reduction of cell viability from 45.87% to 61.13%, which means that all tested materials induce a cytotoxic effect on fibroblasts. In control groups the reduction of viability was 4.56–6.16%.

The percentage of necrotic cells caused by tested adhesives was different. All differences were statistically significant; the levels of differences are presented in Table 3.

The modified apoptotic index for BLEND-A DENT Plus was 45.87, for PROTEFIX it was 52.70 and for COREGA Extra Strong it was 61.13. 

The risk of detecting necrotic cells for all tested adhesives are presented in Table 4. In each case we assessed the risk of detecting necrotic cells in the study group for each dental adhesive compared to the control group. Results were expressed as the odds ratio (OR) with a 95% confidence interval (95% CI) at significance level *p*. The analysis revealed a higher risk for OR > 0, lower risk for OR < 0 and no risk for OR = 0. With regard to the control group the highest risk of detecting necrotic cells was for COREGA Extra Strong and the lowest for BLEND-A-DENT Plus.

Table 5 presents a comparison of odds ratio for detecting necrotic cells between adhesives. The risk of detecting necrotic cells was 1.74 times higher for COREGA Extra Strong than for BLEND-A-DENT Plus and 1.38 times higher than for PROTEFIX. Comparing PROTEFIX and BLEND-A-DENT Plus, the risk was 1.26 times higher for the first adhesive.

The classification of adhesives tested is presented in Table 6. For BLEND-A-DENT Plus in 11 cases the number of necrotic cells was lower than for PROTEFIX and COREGA Extra Strong, and only in 1 case the number of necrotic cells was higher than in PROTEFIX and COREGA Extra Strong. For PROTEFIX, in 3 cases the number of necrotic cells was lower than in COREGA Extra Strong and BLENDA-A-DENT Plus, and in 3 cases the number of necrotic cells was higher than for both the other adhesives. For COREGA Extra Strong, there was no case in which the number of necrotic cells was lower than in PROTEFIX and BLEND-A-DENT Plus, and in 10 cases the number of necrotic cells was higher than for both the other adhesives. CLASS 2 means that the tested adhesive compared with the one product induced more necrotic cells and compared to the second, less. In this classification BLEND-A-DENT has the highest number of cases in CLASS 1, which means the lowest cytotoxic effect, and COREGA Extra Strong has the highest number of cases in CLASS 3, which means the highest cytotoxic effect. 

Table 7 presents the values of the chi^2^ Pearson test and Spearman’s rank correlation test for (r) compared pairs of adhesives.

### 3.2. Qualitative Evaluation of Cytotoxic Effect

**Figure 1:**
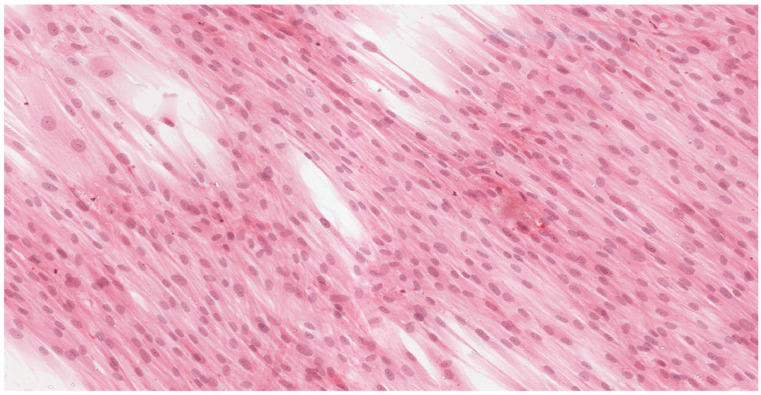
Image of control culture (K); 100× magnification.

**Figure 2:**
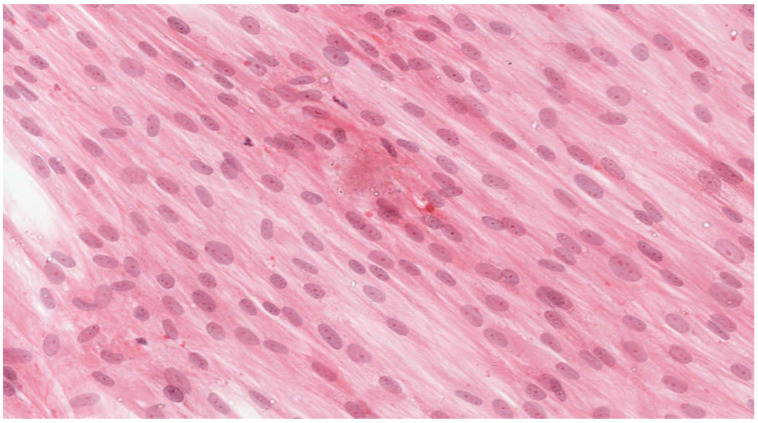
Image of control culture (K); 200× magnification.

**Figure 3:**
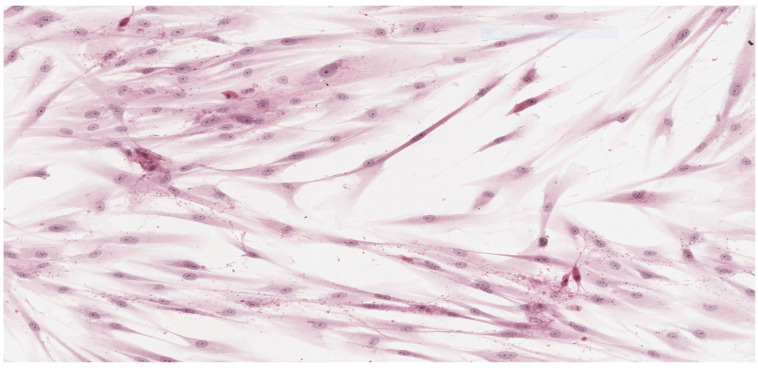
Image of cells cultured on medium with BLEND-A-DENT Plus; 100× magnification.

**Figure 4:**
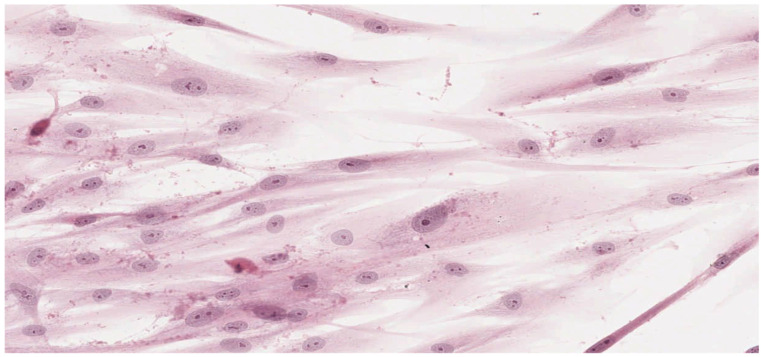
Image of cells cultured on medium with BLEND-A-DENT Plus; 200× magnification.

**Figure 5:**
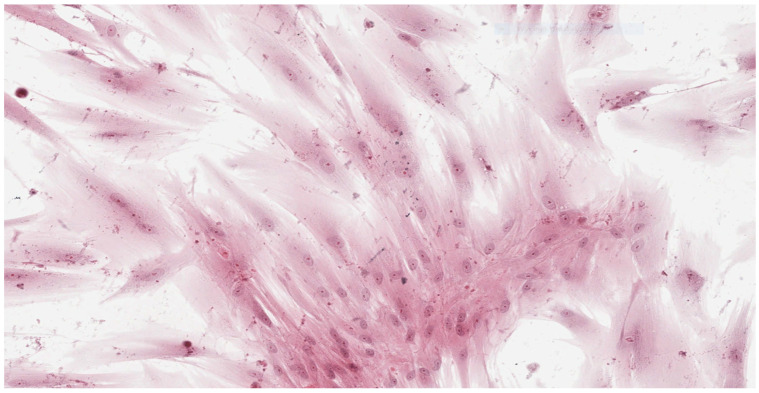
Image of cells cultured on medium with PROTEFIX; 100× magnification.

**Figure 6:**
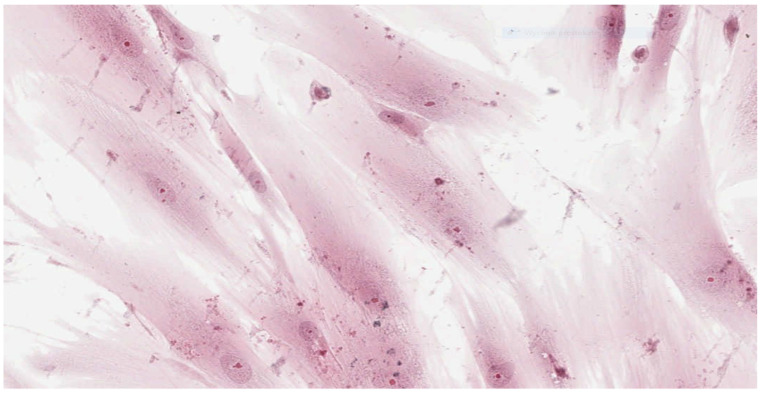
Image of cells cultured on medium with PROTEFIX; 200× magnification.

**Figure 7:**
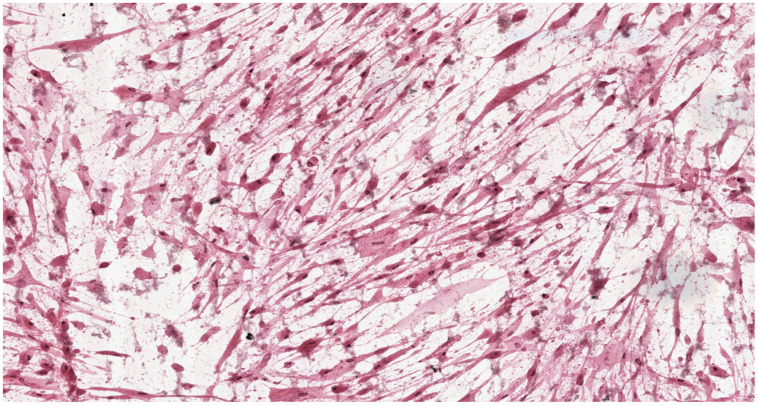
Image of cells cultured on medium with COREGA Extra Strong; 100× magnification.

**Figure 8:**
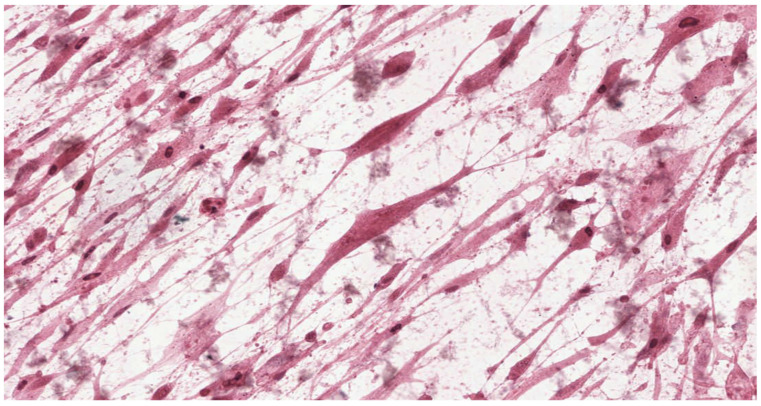
Image of cells cultured on medium with COREGA Extra Strong; 200× magnification.

Analysis of the histopathologic image indicated a small number of degenerative changes in fibroblasts cultured with BLEND-A-DENT Plus. Observation of fibroblasts cultured with COREGA Extra Strong showed the highest diversity of damage and a higher severity of cell damage. In fibroblasts cultured with PROTEFIX signs of cell damage were moderate. The histopathologic images of control cells culture and cells cultured with the tested materials are presented in Figure 1, Figure 2, Figure 3, Figure 4, Figure 5, Figure 6, Figure 7 and Figure 8.

Figure 1 and Figure 2 show the histopathologic images of control cells culture (K) at 100× and 200× magnifications. It is a homogeneous population of proliferating spindle-shaped fibroblasts with tapering ends of the cells; there is no cell lysis and no reduction of cell growth. Oval nuclei can be in the central part of the cell with distinct ruby nucleoli. Intense cytoplasmic staining indicates active protein synthesis. Numerous visible shape changes occurred during mitosis. This image represents grade 0 (no reactivity) in qualitative morphological grading of cytotoxicity according to INTERNATIONAL STANDARD ISO 10993-5:2009(E).

Figure 3 and Figure 4 show the histopathologic images of cells cultured with BLEND-A-DENT. No more than 20% of cells show changes in morphology. Spindle-shaped cells have obvious morphological features of damage. The pale cytoplasm is weakly stained, the cells lose their spindle shape, and the cell margins are blurred. Fibroblasts have different morphology, some with nuclei clearly displaced to one of the ends of the cell. Damaged fibroblasts are malformed and show different cytoplasm eosinophilicity. The number of cells is markedly reduced compared to the control culture. Cellular debris (fragments of disintegrated cells) is seen in the background of the image. This image corresponds to grade 1 (slight reactivity) of qualitative morphological grading of cytotoxicity. 

Figure 5 and Figure 6 show the histopathologic images of cells cultured with PROTEFIX. The changes in morphology are visible in 30% of cells, which do not have a typical spindle shape, and the cell margins are uneven and jagged. Nuclei are absent in some cells, others have pale nuclei without nuclear membrane (cariolysis), which reflects leakage of their contents into the cytoplasm. Cellular debris (fragments of disintegrated cells) is seen in the background of the image. These features indicate necrosis of fibroblasts. This means grade 2 (mild reactivity) cytotoxicity.

Figure 7 and Figure 8 show the histopathologic images of cells cultured with COREGA Extra Strong. Fibroblasts demonstrate morphological features of acute damage. All cells are markedly malformed due to loss of cell membrane. There is a lack of integrity between cells. Nuclei are absent in most of the damaged fibroblast, others present with a disintegrating nucleus. Cytoplasm is excessively eosinophilic. Cellular debris (fragments of disintegrated cells) is seen in the background of the image. The changes are observed in more than 70% of cells, therefore, it can be concluded grade 3 (moderate reactivity) cytotoxicity exists.

## 4. Discussion

The presented study analyzed the biocompatibility of three denture adhesives. The cytotoxicity of the adhesives was assessed in an assay with fibroblasts extracted from mature permanent human teeth, a model reflecting the effect of denture adhesives on fibroblasts from oral tissues. Mesenchymal-derived connective tissues including heart, lung, gastrointestinal tract and muscle contain fibroblasts that fulfill specialized functions [25,26,27,28,29,30]. Differences in gene expression have been demonstrated between dermal and nondermal fibroblasts, and fibroblasts derived from different anatomical sites have differing developmental origins, including the neural crest, lateral plate mesoderm and dermatomyotome [31]. Some studies on fibroblasts from different anatomical sites found marked topographic differences in expression of genes related to growth and differentiation, ECM production, cell migration, lipid metabolism and various genodermatoses, which are molecularly regulated [27,34], but the reaction on toxic materials is similar, regardless of the place of origin. There are a lot of studies that have evaluated the effect of dental materials not having contact with gingiva on gingival fibroblasts [35,36,37,38,39]. Thus, an assumption was made that all fibroblasts from oral tissues follow the same metabolic traits, and for the experiment, dental pulp fibroblasts were used. 

After a predefined culture time the rates of viable and necrotic cells were estimated. For all assays using cultured cells as a model system, it is valuable to know how many live and dead cells are present during or after the end of the experiment. Commonly used direct methods of estimating dead cells take advantage of the loss of membrane integrity and the ability of indicator molecules to partition into a compartment, which is not achievable if the cell membrane is intact. The selective staining of dead cells with trypan blue and microscopic examination is one of the most frequently used routine methods to determine the cell number and percent viability in a population of cells. Viable cells have a clear cytoplasm, whereas dead cells have a blue cytoplasm. All tested adhesives demonstrated a significantly higher amount/percentage of necrotic fibroblasts compared to controls, which testifies to their cytotoxic effect. The adhesives differed regarding their cytotoxic potential. The weakest negative effect was found for BLEND-A-DENT Plus, and the strongest for COREGA Extra Strong. PROTEFIX demonstrated a moderately toxic effect on cell cultures. 

There is a limited number of reports on the cytotoxicity of denture adhesives. Papers published concern COREGA Extra Strong and PROTEFIX [18,19,20]. However, we found no studies investigating the effects of BLEND-A-DENT Plus. Results reported by other researchers seem to be consistent with those presented in our paper, despite the use of different types of tests evaluating cell viability. Depending on the method used, the toxicity of the tested adhesives was defined as mild to moderate. Ekstrand et al. [40] reported that in addition to the lysis of cultured cells, samples showed microbial growth despite the addition of antibiotics to growth media, indicating microbial contamination of denture adhesives. Other researchers [18] reported that denture adhesives, including PROTEFIX, showed significantly stronger cytotoxicity compared to the controls in the MTT assay (colorimetric assay for assessing cell metabolic activity) and in the flow cytometric apoptosis assay. Yamada et al. studied the cytotoxicity of six denture adhesives in direct and indirect human epidermal keratinocyte cells and human oral fibroblasts cultures [41]. They observed the cytotoxicity of all tested materials in both cell culture systems and suggested patients should be careful regarding overuse of denture adhesives in terms of amount and duration.

On the other hand, Al et al. [42] found no cytotoxic effect of PROTEFIX on murine fibroblasts in the MTT assay. The inconsistency of the results may be attributed to different species (human and murine) used in the abovementioned studies. Similarly, de Gomes et al. [22] also used MTT assay and cultures of L929 fibroblasts on agar gels containing denture adhesives, including COREGA, and demonstrated its low cytotoxicity. Chen et al. [21] defined the cytotoxic effect of PROTEFIX as mild or moderate, depending on the used culture medium. López-García et al. evaluated the viability of gingival fibroblasts in the presence of six different denture adhesives using MTT assay [43]. Two of them were equivalent to products evaluated in the present study. Poligrip Flavour Free (GlaxoSmithKline, Consumer Healthcare SA. Stafford-Miller Ireland Ltd., Waterford, Ireland) is an equivalent of COREGA Extra Strong, and Fixodent Pro Plus Duo Protection (Procter & Gamble Portugal S.A., Qta da fonte, Ed. Álvares Cabral, 2774-527, Paço de Arcos, Portugal) is an equivalent of BLEND-A-DENT Plus. They found that denture adhesive containing zinc (Fixodent Pro Plus Duo Protection) could be responsible for the decrease of cell viability and aberrant cell morphology as well as induction of apoptosis and cell death. Our study provided contrary results; the necrosis induced by zinc containing BLEND-A-DENT Plus was lower than that induced by zinc-free PROTEFIX and COREGA Extra Strong. The differences between our observations and those made by López-García et al. seem interesting, but require further research, since other components in denture adhesives might be responsible for cell apoptosis. After all, zinc has been used for a very long time as a therapeutic agent in skin and wound care. Rembe at al. showed relevant pro-proliferative, antimicrobial and tendential anti-apoptotic properties of zinc derivatives in an in vitro study [44].

Results obtained from laboratory cultures and viability evaluation of cells are supported by findings from microscopic analysis of morphological changes. Pathomorphological assessment suggests a lower degree of damage to the morphology of fibroblasts in samples with BLEND-A-DENT Plus—grade 1 cytotoxicity with slight reactivity—and the highest in samples with COREGA Extra Strong—grade 3 cytotoxicity with moderate reactivity. The authors found no publications describing the results of similar studies. 

This study demonstrated differences in the cytotoxic effect of three denture adhesives on fibroblasts. This may be caused by potentially toxic ingredients. Researchers have attributed this effect to different ingredients [20,21]: formaldehyde is associated with cytotoxic and allergenic effects, whereas karaya gum reduces pH below the critical value for enamel [23]. A similar potential has also been reported for antibacterial and antifungal compounds of adhesive creams [15,16]. It is difficult to identify any specific factor responsible for the adverse effects reported because detailed information regarding the composition and concentration of individual ingredients of adhesives is rarely provided by manufacturers.

The composition of three analyzed denture adhesives is similar but not identical. The most important difference refers to the preservatives. Perhaps the different cytotoxic effect on pulp fibroblasts may be due to the content of different preservatives. Research has shown that propylparaben exerts a cytotoxic effect on human fibroblasts in vitro [45]. It serves as an antifungal and an antimicrobial agent. Corega Extra Strong containing propylparaben demonstrated in this study the strongest toxicity. Protefix contains methyl benzoate, a substance that kills or slows the growth of microorganisms, including bacteria, viruses, fungi and protozoans. Methyl benzoate seems to be less cytotoxic than propylparaben, but the authors did not find any relevant comparative study. In an in vitro study Bunch et al. found that methyl benzoate made cells less viable, but they grew well compared to the control [46]. Thus, the cytotoxic effect was considered as minimal. The manufacturer of BLEND-A-DENT Plus does not provide any preservative, and this adhesive demonstrated the lowest cytotoxic effect compared to the other two tested materials. Perhaps the cause of the cytotoxicity is not the zinc content, but the preservatives. This requires clarification in further research. 

In 2010 the European Union Scientific Committee on Consumer Safety stated that the use of butylparaben and propylparaben as preservatives in finished cosmetic products may be considered safe to the consumer, as long as the sum of their individual concentrations does not exceed 0.19% [47]. 

It is clear that many other materials or drugs may have an effect on the oral mucosa, either directly or indirectly through biofilm formation [48]. Further research in the field of the cytotoxic effects of various dental materials could be focused on stem cells, which can be isolated from oral tissues and contribute to their regeneration [49]. Another important issue for future research could be the effects of lasers used in dentistry on oral cells, since laser therapy has gained an important role in contemporary dental therapy [50,51].

Possible limitations of the present study may be associated with its in vitro design, duration and concentration. In vitro studies carried on various cell types (human epidermal keratinocyte cells, human oral fibroblasts cultures, gingival fibroblasts) have shown the cytotoxic effect of adhesive creams, as shown by the results of this study. It can be suspected that the use of denture adhesives may cause cellular damage in human fibroblasts in vivo resulting in adverse health effects. The manufacturers’ recommendations regarding the amount of the product used are intended to prevent exceeding the permissible doses of any ingredients. However, the observations show that patients use too much of denture adhesives and for an extended time period, which may have undesirable effects.

Thus, dentists should advise patients not to overuse denture adhesives, both in terms of product quantity applied and using time. We also suggest that the use of these products should be limited only to cases where the denture does not show proper retention and only in exceptional situations. After all, there is a need for in vivo studies in this field.

## 5. Conclusions

All the three adhesive creams analyzed, PROTEFIX, COREGA Extra Strong and BLEND-A-DENT Plus, demonstrated slight to moderate toxic effects on human fibroblasts in in vitro quantitative and qualitative evaluation. The strongest toxicity was demonstrated by COREGA Extra Strong and the weakest by BLEND-A-DENT Plus. In vivo observations are needed to find out if denture adhesives cause a cytotoxic effect in patients. 

## Figures and Tables

**Table 1:** Denture adhesives tested.

Denture Adhesive	Manufacturer	Composition
COREGA Extra Strong	GlaxoSmithKline, Consumer Healthcare SA. Stafford Miller (Ireland) Limited, Clochreane, Youghal Road, Dungarvan, Co Waterford, Ireland	calcium/sodium PVM/MA copolymer, petrolatum, cellulose gum (carboxymethyl cellulose), paraffinum liquidum, propylparaben, aroma, Cl 45430 (erythrosine)
PROTEFIX	Queisser Pharma GmbH&Co. KG, Schleswiger Straße, Flensburg, Germany	calcium/sodium PVM/MA copolymer, carboxymethyl Cellulose, paraffinum, petrolatum, silicon dioxide, menthol, azorubine, methyl benzoate
BLEND-A-DENT Plus	Procter & Gamble GmbH, Sulzbacher Straße, Schwalbach am Taunus, Germany	calcium/zinc PVM/MA copolymer, paraffinum liquidum, petrolatum, cellulose gum (carboxymethyl cellulose), silica, CI 15985 (Yellow 6), menthyl lactate, aroma, CI 45410 (phloxin B), sodium saccharin, limonene, cinnamal, eugenol

**Table 2:** Descriptive statistic for the value of necrotic fibroblasts and differences between study and control groups analyzed using Mann-Whitney test.

Group	Necrotic Cells %							*p*
	Mean	SD	Min.	Max.	Q25	Median	Q75	
PROTEFIX	52.70	7.89	40.44	64.18	45.58	54.21	60.65	<0.0001
K	5.10	2.65	2.59	10.68	3.00	4.41	5.83	
COREGA Extra Strong	61.13	4.02	54.99	69.30	58.89	60.11	63.39	<0.0001
K	6.16	2.82	3.06	10.58	4.00	4.95	9.44	
BLENDA-A-DENT Plus	45.87	5.58	36.44	56.76	42.19	45.07	48.82	<0.0001
K	4.56	1.69	2.42	7.80	3.58	4.00	5.90	

**Table 3:** Significance levels of differences between percentages of necrotic cells in Mann-Whitney test.

Compared Adhesives	*p*
PROTEFIX vs. COREGA Extra Strong	0.0058
PROTEFIX vs. BLENDA-A-DENT Plus	0.0274
COREGA Extra Strong vs. BLENDA-A-DENT Plus	<0.0001

**Table 4:** Risk of detecting of necrotic cells in study groups versus control groups.

Necrotic Cells	OR	95% CI		*p*
BLEND-A-DENT Plus vs. K	17.19	17.12	17.27	<0.0001
PROTEFIX vs. K	19.44	19.35	19.52	<0.0001
COREGA Extra Strong vs. K	23.16	23.07	23.26	<0.0001

OR (odds ratio)—relative risk; 95% CI—95% confidence interval; *p*—significance level.

**Table 5:** Risk of detecting of necrotic cells for different adhesives.

Necrotic Cells	OR	95% CI	*p*
COREGA Extra Strong vs. BLEND-A-DENT Plus	1.74 1.73	1.75	<0.0001
COREGA Extra Strong vs. PROTEFIX	1.38 1.38	1.39	<0.0001
PROTEFIX vs. BLEND-A-DENT Plus	1.26 1.25	1.26	<0.0001

**Table 6:** Classification of denture adhesives BLEND-A-DENT Plus, PROTEFIX and COREGA Extra Strong for their cytotoxic effect.

Adhesive	Number of Classified Cases			
	CLASS 1	CLASS 2	CLASS 3	Total
BLEND-A-DENT Plus	11	2	1	14
	78.57%	14.29%	7.14%	
PROTEFIX	3	8	3	14
	21.43%	57.14%	21.43%	
COREGA Extra Strong	0	4	10	14
	0.00%	28.57%	71.43%	
Total	14	14	14	

**Table 7:** Statistics for comparisons between adhesives.

Adhesive	chi^2^	df	*p*	r	t	*p*
BLEND-A-DENT Plus vs. COREGA Extra Strong	19.03	2	<0.0001	−0.81	6.939	<0.0001
PROTEFIX vs. COREGA Extra Strong	8.10	2	0.0174	0.54	3.244	0.0032
BLEND-A-DENT Plus vs. PROTEFIX	9.17	2	0.0102	−0.53	3.226	0.0038
